# Death of a London Hospital Foundress

**Published:** 1912-12-28

**Authors:** 


### Death of a London Hospital Foundress.

### THE LATE MADAME ANGIOLA ORTELLI.

The Italian Hospital in Queen Square, Bloomsbury, has sustained an irreparable loss through the recent death of Madame Angiola Ortelli, who, in conjunction with her late husband, the Commendatore Ortelli, founded and erected the present hospital. She was a generous benefactress to the hospital, and devoted much of her time to its work. She was also a very liberal supporter of the Italian Church and School, indeed of every philanthropic effort for the Italian colony in London.

At a meeting held at the hospital on the 6th inst. the Italian Ambassador, the Marchal Imperiali, who presided, moved a vote of condolence with the family. His Excellency said that Madame Angiola Ortelli gave the fullest and most practical expression to her name, for she had for years past proved herself a veritable guardian angel of the poor Italians in London. She had built the beautiful Italian Hospital, never resting until she had perfected all its equipment.

Mr. Stuart Coats, the Chairman of the Hospital Committee, referred to some incidents in her life, and stated that she and her husband had been inspired by pity to provide the hospital for the treatment of sick and destitute Italians stranded in a foreign land. Signor Ortelli died in 1898, after the plans of the present hospital had been prepared, and his widow had spared neither money nor energy fully to carry out their pious intentions. He was able to announce that Madame Ortelli had bequeathed the sum of £10,000 to the institution. Lord Edmond Talbot, M.P., the Vice-Chairman, also referred to Madame Ortelli's strenuous life for others, which, he said, should be an example to all. She was a woman of keen intellect and of large sympathies. As an Englishman he was glad to have this opportunity of expressing his gratitude on behalf of numbers of poor English and Irish Catholics who participated with Italians in the benefits of the hospital. A requiem mass was sung in the Italian Church on the 11th inst., and was attended by the Italian Ambassador. There was a very large congregation, including representatives of the Committee and staff of the Italian Hospital, the Italian Chamber of Commerce, the schools, and by both rich and poor members of the Italian colony in London. The body was afterwards transported to Appiano, near Como, where it will rest in the family vault beside that of her husband.

